# Comparative Chromosome Painting Clarifies the Intraspecific Chromosomal Variation in Two *Ctenomys* Species (Rodentia: Ctenomyidae)

**DOI:** 10.3390/ani15213091

**Published:** 2025-10-24

**Authors:** Thays Duarte de Oliveira, Natasha Ávila Bertocchi, Luciano Cesar Pozzobon, Ivanete de Oliveira Furo, Edivaldo Herculano Corrêa de Oliveira, Jorge C. Pereira, Malcolm A. Ferguson-Smith, Rafael Kretschmer, Thales R. O. de Freitas

**Affiliations:** 1 Departamento de Zoologia, Instituto de Biociências, Universidade Federal do Rio Grande do Sul, Porto Alegre 91501-970, Rio Grande do Sul, Brazil; thaysbiotec@gmail.com; 2 Departamento de Genética, Instituto de Biociências, Universidade Federal do Rio Grande do Sul, Porto Alegre 91501-970, Rio Grande do Sul, Brazil; bertocchinatasha@gmail.com (N.Á.B.); lcpozzobon48@gmail.com (L.C.P.); rafael.kretschmer@ufrgs.br (R.K.); 3 Coordenação de Biodiversidade (COBIO), Instituto Nacional de Pesquisas da Amazônia, Manaus 69060-001, Amazonas, Brazil; ivanete.furo@inpa.gov.br; 4 Instituto de Ciências Exatas e Naturais, Universidade Federal do Pará, Belém 66075-110, Pará, Brazil; ehco@ufpa.br; 5 Laboratório de Cultura de Tecidos e Citogenética, SEAMB, Instituto Evandro Chagas, Ananindeua 67030-000, Pará, Brazil; 6 Animal and Veterinary Research Centre (CECAV), University of Trás-os-Montes and Alto Douro (UTAD), 5000-801 Vila Real, Portugal; jorgecpereira599@gmail.com; 7 Genetics4U, University of Trás-os-Montes and Alto Douro, 5000-801 Vila Real, Portugal; 8 Cambridge Resource Centre for Comparative Genomics, Department of Veterinary Medicine, University of Cambridge, Cambridge CB3 0ES, UK; maf12@cam.ac.uk

**Keywords:** rearrangements, chromosomes, FISH, Hystricomorpha, tuco-tuco, rodent chromosomes, chromosomal evolution

## Abstract

**Simple Summary:**

*Ctenomys minutus* and *Ctenomys lami* are rodents known for their karyotypic diversity. The *C. minutus* diploid number ranges from 2*n* = 42–50, divided into seven parental cytotypes. While *C. lami* diploid number ranges from 2*n* = 50–58, it is divided into four parental cytotypes. To understand the chromosomal evolution of these species, we used whole-chromosome painting with chromosomic probes from *Ctenomys flamarioni*. We identified chromosomal rearrangements shared among *C. minutus* and *C. lami*, and within different cytotypes from within each species. Moreover, several conserved chromosomes across all three *Ctenomys* species were observed. These rearrangements shed light on the chromosomal evolution of the genus *Ctenomys*, particularly within the *torquatus* group, suggesting that these rearrangements have a role on diversification.

**Abstract:**

Background: *Ctenomys* is a subterranean rodent genus known for exhibiting the highest levels of chromosome variation, both among species (2*n* = 10 to 70) and within species. *Ctenomys minutus* is particularly notable for its extensive chromosomal diversity, comprising the greatest number of described cytotypes within this genus. In contrast, *Ctenomys lami* presents the highest degree of karyotypic variation within a comparatively restricted geographic range. Both species inhabit the coastal plain of southern Brazil: *C. minutus* occurs in dunes and sandy fields extending from Laguna (Santa Catarina State) to São José do Norte (Rio Grande do Sul State), whereas *C. lami* is restricted to the “Coxilha das Lombas” region, which lies parallel to the distribution of *C. minutus* in Rio Grande do Sul State. Despite their close evolutionary relationship and the absence of external morphological differences, the mechanism underlying their karyotypic divergence remains poorly understood. Methods: In this study, we applied whole-chromosome painting using probes from *Ctenomys flamarioni* to investigate chromosomal evolution in *C. minutus* and *C. lami*. Results: The resulting homology maps revealed a variety of chromosomal rearrangements that differentiate cytotypes both within and between these species. Comparative analyses demonstrated substantial karyotypic divergence from *C. flamarioni*, although some entire chromosomes and large chromosomal segments remained conserved between *C. minutus* and *C. lami.* Our findings underscore the critical role of chromosomal rearrangements in shaping the diversification of *Ctenomys*. Additionally, we identified shared chromosomal rearrangements in *C. minutus* and *C. lami*, which are likely restricted to the *torquatus* group. Conclusions: These rearrangements provide new insights into the processes driving chromosomal evolution in genus *Ctenomys*.

## 1. Introduction

Chromosomes are fundamental to the structural organization of genomes, making chromosomal studies crucial for deciphering evolutionary trajectories and understanding karyotype differentiation [1,2,3,4,5,6]. Numerous speciation models suggest chromosomal rearrangements can act as barriers to gene flow, reducing genetic exchange between populations (review in [7]). Additionally, these rearrangements may contribute to the preservation of co-adapted gene complexes by inhibiting recombination within the affected chromosomal regions [7,8,9].

Most taxa undergo minimal karyotype changes during speciation, and their karyotypes typically remain conserved at the genus or family level, as seen in Felidae species [10]. However, some groups exhibit considerable interspecific karyotypic variation. *Ctenomys* is particularly notable among mammals for its exceptional chromosomal diversity, with diploid numbers (2*n*) ranging from 2*n* = 10 to 70 [11]. Furthermore, species within this genus, such as *Ctenomys pearsoni*, *Ctenomys minutus*, and *Ctenomys lami*, also show significant interspecific variation [12,13,14].

*Ctenomys* is the most diverse genus of subterranean rodents, comprising at least 65 species [15]. Phylogenetically, it is divided into eight monophyletic groups: *boliviensis*, *frater*, *mendocinus*, *opimus*, *magellanicus*, *talarum*, *torquatus*, and *tucumanus* [16]. Among these, the torquatus group, which includes eight species, exhibits the second-highest level of karyotypic variation within the genus. Diploid numbers ranging from 2*n* = 42 to 70 with fundamental numbers (FN) varying from 68 to 84 [17]. Many species in this group are karyotypically polymorphic, displaying multiple cytotypes, primarily due to Robertsonian (Rb) translocations. Notable examples include *Ctenomys torquatus* (2*n* = 40 to 46, FN = 72), *C. pearsoni* (2*n* = 56–70, FN = 76 to 84), *C. lami* (2*n* = 54 to 58), and *C. minutus* (2*n* = 42 to 50) [17]. *C. minutus* and *C. lami* are sister species within the torquatus group and are endemic to the coastal plain of southern Brazil [16,18,19].

Despite their chromosomal differences, *C. minutus* and *C. lami* are externally indistinguishable. *C. lami* was recognized as a distinct species from *C. minutus* based on variations in geographic distribution, karyotype organization, and skull morphology [18]. The karyotypic divergence between these species extends beyond the difference in diploid numbers, encompassing chromosomal rearrangements contributing to intraspecific polymorphism. These rearrangements also lead to distinct chromosomal morphologies, which may be unique for each species [18].

*Ctenomys minutus* is a species endemic to southern Brazil, found explicitly in the dunes and sandy fields along the coastal plains of the Santa Catarina (Laguna) and Rio Grande do Sul States (São José do Norte) [20]. This species is notable for its exceptional karyotypic diversity, with 45 distinct karyotypes described, more than any other species in the genus. These karyotypes result from varying combinations of 2*n* ranging from 42 to 50 and FN from 68 to 80 [14,18,19]. Seven primary cytotypes parapatrically distributed across the region from north to south are identified: 2*n* = 50a, 48a, 46a, 42, 46b, 48b, and 50b—(designated here as I to VII) [14,21]. The presence of four intraspecific hybrid zones has resulted in the formation of intermediate karyotypes, which exhibit combinations of these parental diploid numbers [14,20,21,22,23,24].

Each cytotype of *C. minutus* is linked to a specific geographic area along the coastal plain of southern Brazil, exhibiting either a contiguous distribution or being separated by geographic barriers or paleochannels [25]. The 2*n* = 50 cytotype characterizes the distribution extremes in the North and South. The standard karyotype is the northernmost cytotype (cytotype I-2*n* = 50a), from which diploid numbers gradually decrease to 2*n* = 42 (cytotype IV) due to Robertsonian rearrangements. Conversely, toward the southernmost distribution, the diploid number increases to 2*n* = 50b (cytotype VII). Cytotypes with the same diploid number are differentiated as “a” or “b” based on structural rearrangements in distinct chromosomes, including tandem fusions/fissions, as well as paracentric or pericentric inversions [20,21,22,23,26].

*Ctenomys lami* has a highly restricted geographic range, occupying an area of approximately 78 km × 12 km of sandy fields in the “Coxilha das Lombas” in Rio Grande do Sul State, Brazil [13,18,27]. Despite this limited distribution, it exhibits one of the highest karyotypic variability known within the genus *Ctenomys*, with five distinct diploid numbers (2*n* = 54, 55, 56, 57, and 58) and ten fundamental numbers (FN = 74 to 82 and 84), resulting in a total of 26 different karyotypes within just 936 km^2^ [13,18]. These variations in diploid numbers are primarily due to Robertsonian rearrangements, centric fusions/fissions, and pericentric inversions [13]. As seen in *C. minutus*, cytotypes designated as “a” or “b” differ due to rearrangements that affect different chromosomes [13].

Chromosome painting, using whole-chromosome probes, has revolutionized comparative cytogenetics, especially in rodents, by illuminating regions of homology between species [28]. For example, this technique has elucidated the evolutionary processes in the genus *Ellobius*, which showcases remarkable karyotypic diversity. It has helped to reveal the driving forces behind diversification and speciation in these species [26]. Yet, comparative cytogenetic studies using chromosome painting remain scarce when it comes to *Ctenomys*, a genus with equally intriguing chromosomal variation. While some species have been studied with banding techniques (see [29]), only one investigation applied chromosome painting, focusing on *C. minutus* and *C. flamarioni* [30]. In this study, Kubiak et al. [30] performed chromosome painting on a single cytotype of *C. minutus* (2*n* = 46a—cytotype II) to compare parental karyotypes (*C. flamarioni* and *C. minutus*), as well as their hybrids, shedding light on the origins of these hybrid forms.

Our study builds on this work, offering a deep look into chromosomal evolution within *Ctenomys* by focusing on two closely related species, *C. minutus* and *C. lami*. These species’ striking karyotypic diversity, indistinguishable external appearances, and limited geographic ranges present a fascinating opportunity to explore how chromosomal rearrangements may drive speciation. What is even more exciting is the unique chromosomal traits and intraspecific variability they exhibit. By employing the set of *C. flamarioni* chromosome probes [30], we dive into the chromosomal evolution of both *C. minutus* and *C. lami*.

## 2. Materials and Methods

### 2.1. Sample Collection

**Figure 1:**
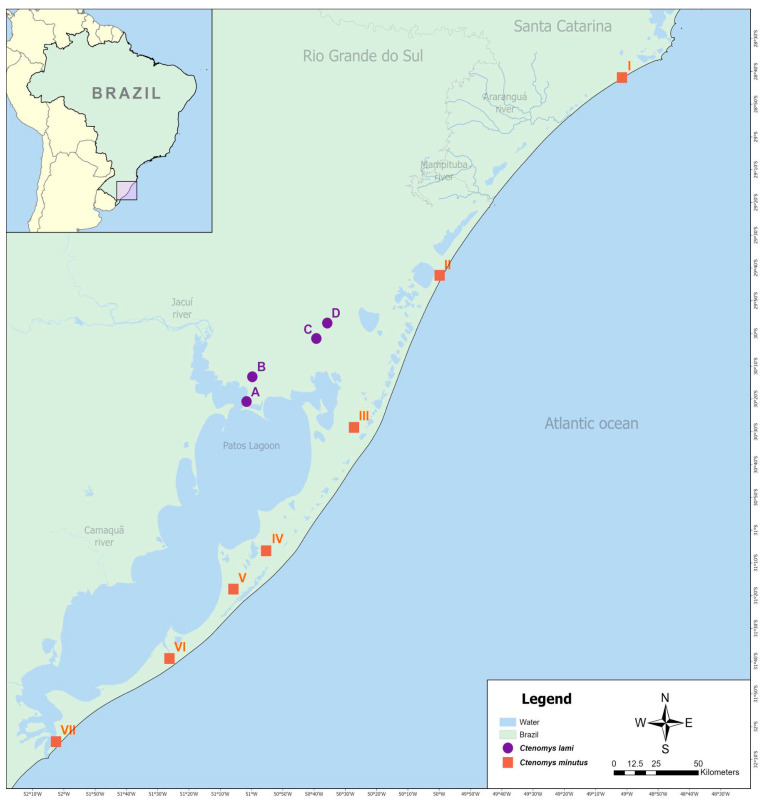
Geographic origin of the *Ctenomys minutus* and *C. lami* specimens analyzed in this study. Map based on the image of the coastal plain of southern Brazil. The circles and the squares indicate the approximate locations of the *C. minutus* and *C. lami* samples, respectively, as listed in Table 1, indicating the Blocks/Cytotypes.

Thirteen *C. minutus* and eight *C. lami* individuals were collected in Rio Grande do Sul and Santa Catarina States, Brazil (Table 1; Figure 1). Animals were captured using Oneida Victor No.0 plot traps and euthanized in accordance with the guidelines of the American Society of Mammalogists’ Animal Care and Use Committee [31]. All procedures were approved by the Ethics Committee for the Use of Animals (CEUA-UFRGS, protocol No. 35828) and authorized by the Brazilian Environmental Agency (IBAMA, approval No. 14690-1).

### 2.2. Chromosomal Preparations and Karyotype

Chromosome preparations were obtained from fibroblast cultures following the method of Verma and Babu [32], with modifications. Briefly, kidney and/or lung biopsies were enzymatically digested with collagenase and cultured at 37 °C in Dulbecco’s Modified Eagle Medium–high glucose (DMEM; GIBCO) supplemented with 20% fetal bovine serum (GIBCO), penicillin (100 U/mL), and streptomycin (100 µg/mL). After a 1h incubation with colchicine, cells were exposed to a hypotonic solution (0.075 M KCl) for 8 min and subsequently fixed in methanol/acetic acid (3:1). For each specimen, ~30 metaphases were examined to determine diploid number and chromosome morphology.

### 2.3. Fluorescence In Situ Hybridization (FISH)

Fluorescence in situ hybridization experiments were carried out on all specimens and cytotypes of *C. minutus* and *C. lami* using whole-chromosome probes from *C. flamarioni* (CFL 1–23 and X), obtained by flow sorting at the Cambridge Resource Centre for Comparative Cytogenetics (Cambridge, UK) [30]. Probes were amplified and labeled with biotin or digoxigenin using DOP-PCR. Hybridization procedures followed Kubiak et al. [30]. Biotin-labeled probes were detected with Cy3-avidin, and digoxigenin-labeled probes with anti-rabbit FITC. Slides were counterstained with Fluoroshield™ containing DAPI (Sigma-Aldrich, St. Louis, MO, USA). Images were captured using a ZEISS Axiophot epifluorescence microscope (Carl Zeiss, Jena, Germany) with ZEN BLUE (Carl Zeiss, Jena, Germany) software (https://www.zeiss.com/microscopy/en/products/software/zeiss-zen.html (accessed on 21 October 2025)) and processed with Adobe Photoshop CS6.

## 3. Results

### 3.1. Karyotypes

The karyotypes observed in our sample of *C. minutus* corresponded to cytotypes 50a, 48a, 46a, 42, 46b, 48b, and 50b, as previously described by Lopes et al. [14], Freygang et al. [20] and Freitas [22]. For *C. lami* individuals, the karyotypes align with those previously described by Freitas [13,18], with individuals exhibiting the cytotypes 2*n* = 54, 2*n* = 56, and 2*n* = 58, captured in blocks A and D, block C, and block B, respectively.

### 3.2. Comparative Chromosome Painting

**Figure 2:**
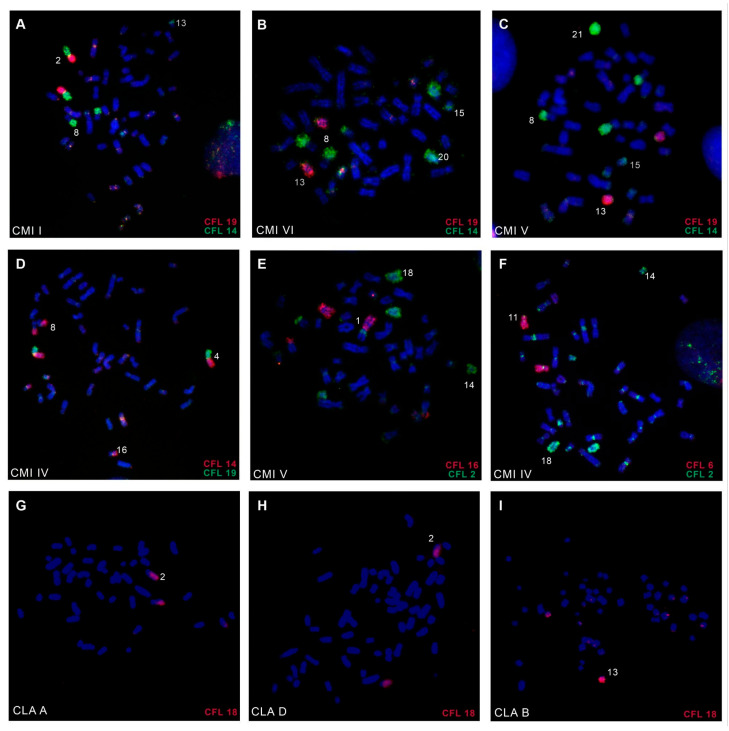
Representative FISH experiments with *C. flamarioni* (CFL) probes (**A**–**F**) in different cytotypes of *C. minutus* an (**G**–**I**) *C. lami. C. minutus:* (**A**) showing fusion of CFL19/14 and fission of CFL14 on cytotype I (2*n* = 50a), (**B**,**C**) showing fission of CFL14 on cytotype VI (2*n* = 48b) and V (2*n* = 46b), (**D**) showing fusion of CFL14/19 on cytotype IV (2*n* = 42) (**E**,**F**) showing fission of CFL2 on cytotype V (2*n* = 46b) and IV (2*n* = 42). *C. lami*: (**G**–**I**) Only homologies are shown. Cytotypes used are indicated in the lower-left corner of the images. The probes used are indicated in the lower-right corner of the images. Chromosomes were counterstained with DAPI (blue), and the probes were labelled with Biotin-CY3 (red) and digoxigenin-FITC (green).

**Figure 3:**
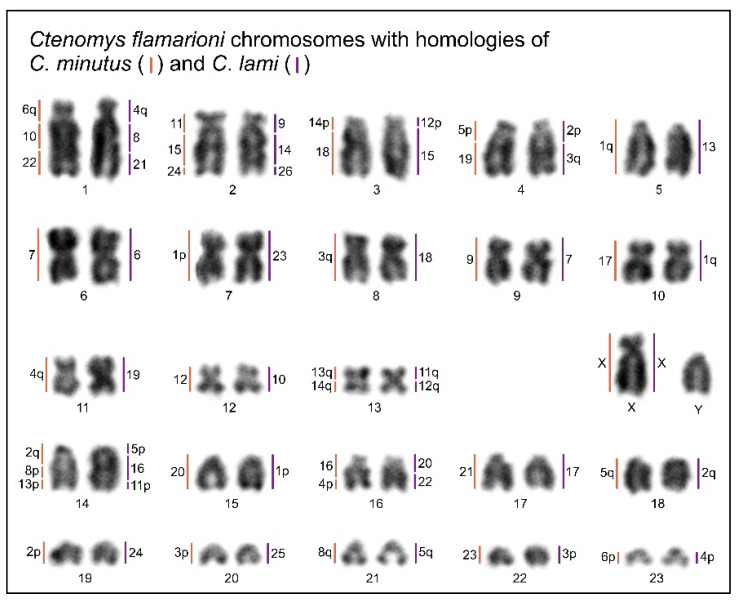
Homology map of standard cytotype of *C. minutus* (Block I—50a; orange) and *C. lami* (Block A—54; purple) with *C. flamarioni.* The number on the right side of each chromosome is the homology with the probes of *C. flamarioni*.

**Figure 4:**
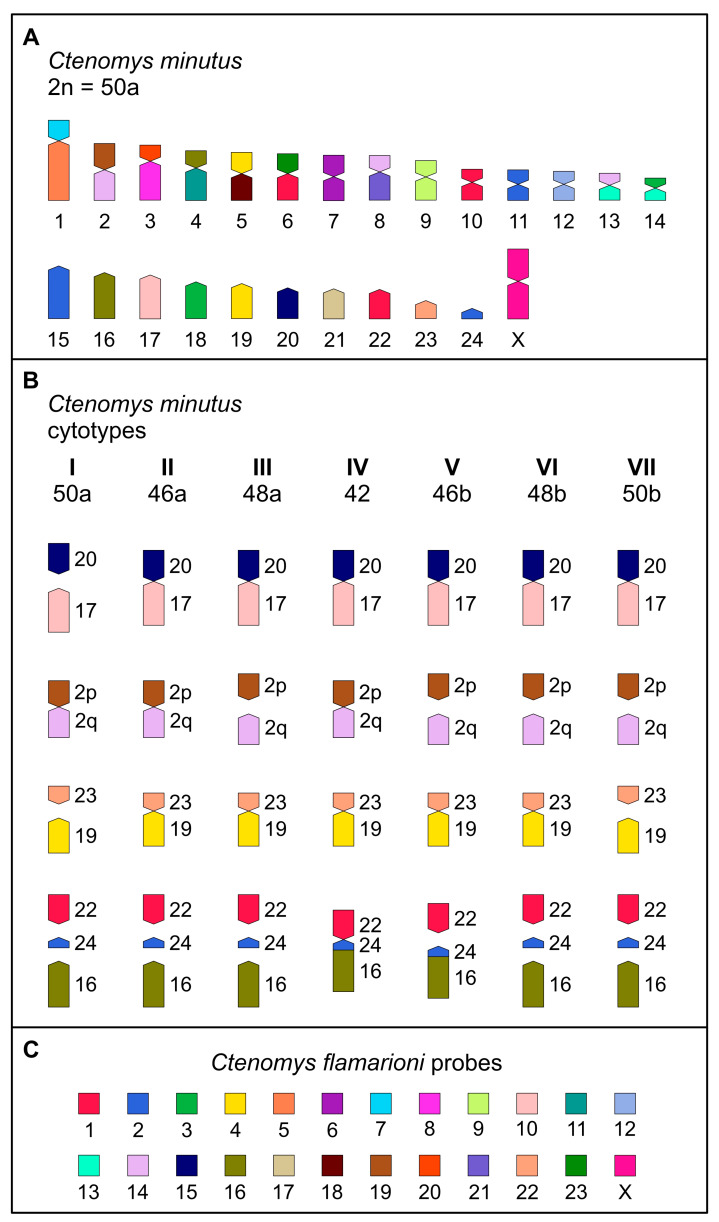
Homology map of *C. minutus* with *C. flamarioni*. (**A**) Homology map of standard cytotype of *C. minutus* (cytotype I—50a) with *C. flamarioni.* (**B**) Representation of the chromosomal variation from cytotypes I to VII of *C. minutus*, the cytotypes are arranged according to their geographic distribution as shown in Figure 1. (**C**) Each colour represents the homologous chromosome of *C. flamarioni*.

Chromosome-specific paints from *C. flamarioni* (CFL), corresponding to all autosomes and the X chromosome, were successfully hybridized to the metaphases of *C. minutus* and *C. lami* (Figure 2). The results allowed for the construction of comparative chromosome maps for all cytotypes of both species, *C. minutus* (CMI) and *C. lami* (CLA) (Table 2; Figure 3 and Figure 4).

Karyotype 50a (cytotype I) is regarded as the standard karyotype for *C. minutus* [22]. Using this reference, we established a comparative homology map between *C. flamarioni* and *C. minutus* (cytotype I; Figure 3 and Figure 4A). In cytotype I, seven autosomes exhibited homology to only one *C. flamarioni* probe: CFL6 with CMI7; CFL9 with CMI9; CFL10 with CMI17; CFL12 with CMI12; CFL15 with CMI20; CFL17 with CMI21; and CFL22 with CMI23. Notably, the homology of CFL10 and CFL15 is exclusive to cytotype I (50a), as in other cytotypes these chromosomes are involved in fusion events. Additionally, CFL19 corresponds to the short arm of chromosome 2 (CMI2p). In cytotypes III, V, VI, and VII, chromosome 2 undergoes fission, resulting in 2p as an independent chromosome, thereby preserving complete homology with CFL19. Apart from these homology regions, the remaining *C. minutus* chromosomes show seven fissions (CFL3 with CMI14p and CMI18; CFL4 with CMI5p and CMI19; CFL13 with CMI13q and CMI14q; CFL14 with CMI2q, CMI8p and CMI13p; and CFL16 with CMI4p and CMI16) and nine fusions (CFL7/5, CFL19/14, CFL 20/8, CFL16/11, CFL4/18, CFL 23/1, CFL14/21, CFL14/13, CFL3/14) (Table 2; Figure 3 and Figure 4).

**Figure 5:**
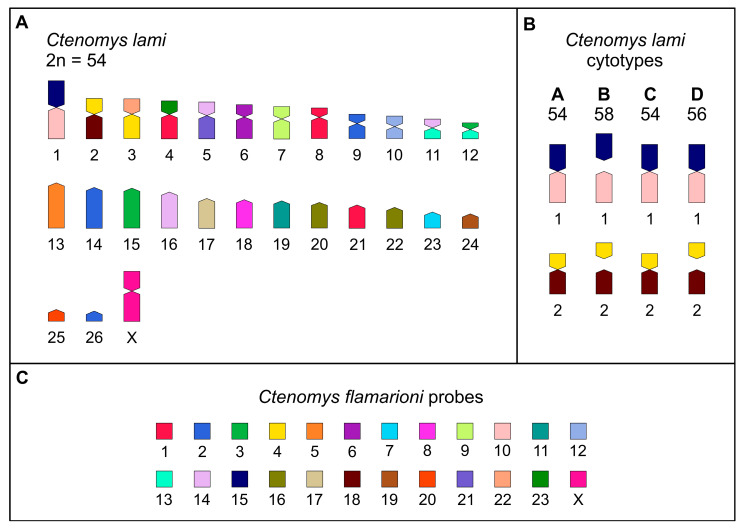
Homology map of *C. lami* with *C. flamarioni*. (**A**) Homology map of standard cytotype of *C. lami* (block A 54) with *C. flamarioni*. (**B**) Representation of the chromosomal variation from cytotypes A to D of *C. lami*, the cytotypes are arranged according to their geographic distribution as shown in Figure 1. (**C**) Each colour represents the homologous chromosome of *C. flamarioni*.

For *C. lami*, cytotype 54 (Block A) is considered the standard karyotype for the species. Based on this reference, we constructed a homology map between *C. flamarioni* and *C. lami* (cytotype Block A; Figure 3 and Figure 5A). Ten *C. lami* autosomes exhibited homology to *C. flamarioni* chromosomes, as follows: CFL5 with CLA13; CFL6 with CLA6; CFL7 with CLA23; CFL8 with CLA18; CFL9 with CLA7; CFL11 with CLA19; CFL12 with CLA10; CFL17 with CLA17; CFL19 with CLA24; and CFL20 with CLA25 (Table 2; Figure 3). In cytotype 56b (Block D) and cytotype 58 (Block B), in addition to these chromosomes, homology was also detected for CFL10 and CFL15, due to the fission of chromosome 1, which gives rise to two distinct chromosomes in these cytotypes. Furthermore, in cytotype 58 (Block B), beyond the homologies identified in cytotype 56b, an additional homology was observed involving CFL18, as a consequence of chromosome 2 fission, which also generates two chromosomes in this block. The remaining *C. lami* chromosomes present seven fissions (CFL3 with CLA12p and CLA15, CFL4 with CLA12p and CLA15, CFL13 with CLA11q and CLA12q, CFL14 with CLA5p, CLA11p and CLA16, and CFL16 with CLA20 and CLA22) and seven fusions: CFL15/10, CLF4/18, CLF22/4, CLF23/1, CLF15/21, CLF14/13, CLF3/13 (Table 2; Figure 3 and Figure 5).

## 4. Discussion

### 4.1. Comparative Chromosome Painting

Cross-species chromosome painting has been applied to the study of karyotypes in more than 100 rodent species (reviewed in [28]). Nevertheless, Zoo-FISH data for the genus *Ctenomys* remain scarce. To date, chromosome-specific probes from *C. flamarioni* have been hybridized exclusively to metaphases of *C. minutus* (cytotype II, 2*n* = 46a), revealing extensive chromosomal rearrangements between *C. flamarioni* and *C. minutus* cytotype II [30]. Our Zoo-FISH experiments provide the first comprehensive comparison of the genome of *C. flamarioni* with the parental cytotypes of *C. minutus* and *C. lami*.

We identified four chromosomes (CFL6, CFL9, CFL12, and CFL17) that are conserved across *C. flamarioni*, all cytotypes of *C. minutus*, and *C. lami* (Figure 3, Figure 4 and Figure 5). In contrast, seven large *C. flamarioni* chromosomes (CFL1, CFL2, CFL3, CFL4, CFL13, CFL14, and CFL16) are fragmented into two or three distinct chromosome pairs in *C. minutus* and *C. lami*, while remaining conserved between these two species (Figure 3). The absence of complete 1:1 synteny supports the hypothesis that karyotypes in *Ctenomys* are highly rearranged [11,33,34,35,36,37], in stark contrast with other rodent genera characterized by highly conserved karyotypes, such as *Peromyscus* (all species with 2*n* = 48) and *Oxymycterus* (all species with 2*n* = 54 and FN = 62–64) [38,39,40].

Multiple chromosomal rearrangements, including fissions and inversions, were detected. Among them were specific rearrangements within *C. minutus* cytotypes, such as the fission and inversion of CMI2p in system “b”, which enabled the reconstruction of the differentiation of cytotypes sharing the same 2n (Figure 3 and Figure 4). Several fusion events were also observed, including CMI17/20 (CFL10 and CFL15), CMI19/23 (CFL4 and CFL22), and CMI22/24/16 (CFL1, CFL2, and CFL16) (Figure 3 and Figure 5), consistent with the results of Kubiak et al. [30]. In *C. lami*, rearrangements of chromosomes 1 and 2 were evident: CFL10 corresponds to CLA1q, CFL15 to CLA1p, part of CFL4 to CLA2p, and CFL18 to CLA2q (Figure 3). These findings are corroborated by G-banding analyses reported by Freyganag et al. [20] and Freitas [13].

Taken together with previous comparative chromosome painting studies, our results demonstrate that Zoo-FISH provides a resolution of rearrangement patterns comparable to G-banding in *C. minutus* and *C. lami*. Nonetheless, while probes derived from *C. flamarioni* are highly effective for identifying interchromosomal rearrangements and assessing chromosomal variability, they are less suitable for detecting intrachromosomal changes. For this purpose, higher-resolution approaches, such as bacterial artificial chromosome (BAC) probes or other region-specific markers, are more appropriate [41,42,43].

### 4.2. Comparative C. minutus x C. lami

Morphological, molecular, and cytogenetic studies have substantially advanced our understanding of taxonomy, phylogenetic relationships, and karyotypic patterns within the genus *Ctenomys*. Lopes et al. [14] has emphasized the intrinsic association of geographic barriers in the fixation of chromosomal rearrangements in this group, as they are fixated by genetic drift and after the removal of the geographic barrier causes reduced gene flow between different cytotypes. Using mitochondrial DNA from representatives across thirty distinct localities, their study revealed that individuals with a 2*n* = 50a karyotype occupy a basal position, whereas those with 2n = 42 represent a more derived condition within the population.

This striking diversity is closely tied to the complex geography of the region, characterized by a mosaic of lakes, lagoons, rivers, swamps, sandy fields, and dunes that act as natural barriers promoting speciation. However, in certain hybrid zones, contact between recently diverged species enables gene flow among populations, which in turn contributes to the emergence of distinct cytotypes [14,44].

In the present study, by comparing the rearrangements identified in *C. minutus* and *C. lami*, we provide insights into the chromosomal changes involved in their karyotypic differentiation. We demonstrate that shared rearrangements between these two species may be associated with a recent speciation process in which chromosomal changes play a pivotal role, as previously hypothesized by [21]. This view is further supported by the ecological context of these species: the presence of strong geographic barriers is consistent with an allopatric model of speciation, followed by the accumulation of diverse chromosomal rearrangements [13,21].

Our data support the hypothesis that *C. lami* (2*n* = 58) possesses a karyotype more similar to the putative common ancestor of the two species. From an evolutionary perspective, given that fusions are generally more frequent than fissions, karyotypes with higher diploid numbers are typically considered closer to the ancestral state [45]. Accordingly, *C. minutus* (2*n* = 42) would represent a more derived condition, characterized by a reduced diploid number and a higher number of chromosomal rearrangements relative to *C. lami* [13,18]. Nevertheless, establishing which karyotype is ancestral and which is derived remains a challenge due to the rapid and recent divergence observed within *Ctenomys* [16].

Overall, our findings provide a framework for investigating chromosomal evolution in *Ctenomys* and can be extended to other species within the genus. Such approaches may contribute to species delimitation, particularly in groups from central-western Brazil. Future studies should combine comparative chromosome painting with whole-genome sequencing to achieve higher-resolution analyses, as has been successfully demonstrated in other taxa [29,46,47].

## 5. Conclusions

In this study, we provide a comparative chromosomal painting analysis of two endemic tuco-tuco species from southern Brazil, *C. minutus* and *C. lami*, which display some of the greatest karyotypic diversity described within the genus. Our results reinforce the hypothesis that chromosomal rearrangements have played a role in the diversification and speciation of *Ctenomys*. We demonstrate that *C. minutus* and *C. lami* share specific chromosomal rearrangements.

Beyond the interspecific comparison, our data contribute to reconstructing the evolutionary dynamics of *Ctenomys* karyotypes, highlighting the importance of fusions and fissions in shaping their remarkable chromosomal variation. Expanding comparative cytogenetic analyses to additional species will be essential for elucidating the broader evolutionary history of the genus.

Finally, integrating classical and molecular cytogenetic techniques with whole-genome sequencing and assembly will provide the resolution needed to detect intrachromosomal rearrangements, uncover the mechanisms driving chromosomal evolution, and clarify the role of these processes in the speciation of *Ctenomys*.

## Figures and Tables

**Table 1:** Number of specimens, sex, and location of each species collected.

Species	Blocks/Cytotypes	Individuals/Sex	Locality	Geographic Coordinate
*C. lami*	A (54)	2 ♀	Parque do Itapuã (RS)	30°20′47.4″ S 51°01′35.6″ W
	B (58)	2 ♀	Passo do Vigário (RS)	30°13′13.1″ S 50°59′43.6″ W
	C (54)	2 ♀	Lombas (RS)	30°01′32.4″ S 50°39′16.6″ W
	D (56b)	2 ♀	Chico Lomã (RS)	29°56′49.8″ S 50°35′48.7″ W
*C. minutus*	I (50a)	1 ♀	Jaguaruna (SC)	28°41′53.02″ S 49°01′33.86″ W
	II (48a)	2 ♀	Praia do barro (RS)	29°42′14.86″ S 49°58′51.86″ W
	III (46a)	2 ♂♀	Bacupari (RS)	30°28′41.01″ S 50°27′13.92″ W
	IV (42)	2 ♀	Mostardas (RS)	31°06′ 17″ S 50°55′20″ W
	V (46b)	2 ♂♀	Tavares (RS)	31°17′ 58,9″ S 51°05′47,6″ W
	VI (48b)	2 ♀	Bojuru (RS)	31°39′10.7″ S 51°26′14.8″ W
	VII (50b)	2 ♀	São José do Norte (RS)	32°04′34.47″ S 52°02′31.47″ W

**Table 2:** *C. flamarioni* probes and its homologous regions in the standard karyotypes of *C. minutus* (2*n* = 50a) and *C. lami* (2*n* = 54).

*C. flamarioni* (2*n* = 48)	*C. minutus* I (2*n* = 50a)	*C. lami* A (2*n* = 54)
1	6q, 10, 22	4q, 8, 21
2	11, 15, 24	9, 14, 26
3	14p, 18	12p, 15
4	5p, 19	2p, 3q
5	1q	13
6	7	6
7	1p	23
8	3q	18
9	9	7
10	17	1q
11	4q	19
12	12	10
13	13q, 14q	11q, 12q
14	2q, 8p, 13p	5p, 11p, 16
15	20	1p
16	4p, 16	20, 22
17	21	17
18	5q	2q
19	2p	24
20	3p	25
21	8q	5q
22	23	3p
23	6p	4p
X	X	X

## Data Availability

All original contributions from this study are included in this article. Additional information is available from the corresponding author upon request.
